# Influenza and Tdap Vaccination Coverage Among Pregnant Women — United States, April 2020

**DOI:** 10.15585/mmwr.mm6939a2

**Published:** 2020-10-02

**Authors:** Hilda Razzaghi, Katherine E. Kahn, Carla L. Black, Megan C. Lindley, Tara C. Jatlaoui, Amy Parker Fiebelkorn, Fiona P. Havers, Denise V. D’Angelo, Angela Cheung, Nicholas A. Ruther, Walter W. Williams

**Affiliations:** ^1^Immunization Services Division, National Center for Immunization and Respiratory Diseases, CDC; ^2^Leidos, Atlanta, Georgia; ^3^Division of Bacterial Diseases, National Center for Immunization and Respiratory Diseases, CDC; ^4^Division of Reproductive Health, National Center for Chronic Disease Prevention and Health Promotion, CDC; ^5^Abt Associates, Inc., Atlanta, Georgia.

Vaccination of pregnant women with influenza vaccine and tetanus toxoid, reduced diphtheria toxoid, and acellular pertussis vaccine (Tdap) can decrease the risk for influenza and pertussis among pregnant women and their infants. The Advisory Committee on Immunization Practices (ACIP) recommends that all women who are or might be pregnant during the influenza season receive influenza vaccine, which can be administered at any time during pregnancy (*1*). ACIP also recommends that women receive Tdap during each pregnancy, preferably during the early part of gestational weeks 27–36 (*2*,*3*). Despite these recommendations, vaccination coverage among pregnant women has been found to be suboptimal with racial/ethnic disparities persisting (*4**–**6*). To assess influenza and Tdap vaccination coverage among women pregnant during the 2019–20 influenza season, CDC analyzed data from an Internet panel survey conducted during April 2020. Among 1,841 survey respondents who were pregnant anytime during October 2019–January 2020, 61.2% reported receiving influenza vaccine before or during their pregnancy, an increase of 7.5 percentage points compared with the rate during the 2018–19 season. Among 463 respondents who had a live birth by their survey date, 56.6% reported receiving Tdap during pregnancy, similar to the 2018–19 season (*4*). Vaccination coverage was highest among women who reported receiving a provider offer or referral for vaccination (influenza = 75.2%; Tdap = 72.7%). Compared with the 2018–19 season, increases in influenza vaccination coverage were observed during the 2019–20 season for non-Hispanic Black (Black) women (14.7 percentage points, to 52.7%), Hispanic women (9.9 percentage points, to 67.2%), and women of other non-Hispanic (other) races (7.9 percentage points, to 69.6%), and did not change for non-Hispanic White (White) women (60.6%). As in the 2018–19 season, Hispanic and Black women had the lowest Tdap vaccination coverage (35.8% and 38.8%, respectively), compared with White women (65.5%) and women of other races (54.0%); in addition, a decrease in Tdap vaccination coverage was observed among Hispanic women in 2019–20 compared with the previous season. Racial/ethnic disparities in influenza vaccination coverage decreased but persisted, even among women who received a provider offer or referral for vaccination. Consistent provider offers or referrals, in combination with conversations culturally and linguistically tailored for patients of all races/ethnicities, could increase vaccination coverage among pregnant women in all racial/ethnic groups and reduce disparities in coverage.

An Internet panel* survey was conducted to assess end-of-season influenza and Tdap vaccination coverage estimates among women pregnant during the 2019–20 influenza season; the methods have been previously described (*5*). The survey was conducted during April 2–April 14, 2020, among women aged 18–49 years who reported being pregnant anytime since August 1, 2019, through the date of the survey. Among 18,314 women who were screened, 2,515 were eligible, and of these, 2,268 completed the survey (cooperation rate^†^ = 90.2%). Data were weighted to reflect the age, race/ethnicity, and geographic distribution of the total U.S. population of pregnant women (*5*). Analysis of influenza vaccination coverage was limited to 1,841 women pregnant anytime during October 2019–January 2020. A woman was considered to have been vaccinated against influenza if she reported having received 1 dose of influenza vaccine (before or during her most recent pregnancy) since July 1, 2019. To accommodate the optimal timing for Tdap vaccination during 27–36 weeks’ gestation, analysis of Tdap coverage was limited to women pregnant anytime since August 1, 2019, who had a live birth by their survey date. A woman was considered to have received Tdap if she reported receiving 1 dose of Tdap vaccine during her most recent pregnancy. Among 532 women with a recent live birth, 69 (12.9%) were excluded because they did not know whether they had ever received Tdap (10.3%) or whether they received it during their pregnancy (2.6%), leaving a final analytic sample of 463. The proportion of pregnant women who received both recommended maternal vaccines (i.e., full vaccination) was assessed among 462 women (one respondent reported Tdap but not influenza vaccination status). A difference was noted as an increase or decrease when a percentage-point difference of ≥5 was found between any values being compared.^§^SAS-callable SUDAAN software (version 11.0.1; RTI International) was used to conduct all analyses.

Among 1,841 pregnant women, 61.2% reported receiving 1 dose of influenza vaccine since July 1, 2019, an increase of 7.5 percentage points compared with 53.7% reported for the 2018–19 influenza season; Tdap coverage was 56.6% among women with a recent live birth, similar to that reported for 2018–19 (54.9%) (Table 1) (Figure). Full vaccination was reported by 40.3% of women with a recent live birth overall, but only among 23.0% of Black and 25.4% of Hispanic women. Influenza vaccination coverage was lowest among Black women (52.7%), and Tdap coverage was lowest among Black (38.8%) and Hispanic (35.8%) women. Vaccination coverage was highest among women who reported receiving a provider offer or referral for vaccination (75.2% for influenza and 72.7% for Tdap). Women who had 10 or more provider visits since July 1, 2019, were more likely to have received influenza vaccine (67.5%) than were those with one to five visits (50.6%).

**TABLE 1:** Influenza and Tdap vaccination coverage among pregnant women, by selected characteristics — Internet panel survey, United States, April 2020

Characteristic	Influenza*			Tdap^†^	Both vaccines (full vaccination)	
	No. (weighted %)	% (weighted) vaccinated	No. (weighted %)	% (weighted) vaccinated	No. (weighted %)	% (weighted) vaccinated
**Total**	**1,841 (100)**	**61.2**	**463 (100)**	**56.6**	**462 (100)**	**40.3**
**Age group (yrs)**						
18–24	631 (24.4)	54.6^§^	88 (13.8)	53.4	87 (13.6)	30.6
25–34	861 (55.6)	62.5	253 (61.6)	60.0^§^	253 (61.7)	44.4^§^
35–49^¶^	349 (20.0)	65.8	122 (24.6)	50.1	122 (24.7)	35.3
Race/Ethnicity**						
White, non-Hispanic^¶^	890 (49.7)	60.6	302 (63.7)	65.5	301 (63.6)	46.0
Black, non-Hispanic	323 (19.7)	52.7^§^	52 (13.9)	38.8^§^	52 (14.0)	23.0^§^
Hispanic	436 (23.1)	67.2^§^	60 (14.1)	35.8^§^	60 (14.1)	25.4^§^
Other, non-Hispanic	192 (7.4)	69.6^§^	49 (8.3)	54.0^§^	49 (8.3)	51.0^§^
**Education**						
High school diploma or less	450 (23.4)	45.9^§^	114 (24.3)	45.2^§^	114 (24.4)	25.0^§^
Some college, no degree	287 (15.4)	50.9^§^	72 (15.6)	54.4^§^	72 (15.6)	40.2^§^
College degree (2- or 4-year)	708 (39.7)	68.3	188 (42.1)	62.7	188 (42.2)	47.0
More than college degree^¶^	396 (21.4)	72.2	89 (18.0)	60.0	88 (17.8)	45.2
**Marital status^††^**						
Married^¶^	1,012 (57.4)	70.3	293 (62.6)	65.3	293 (62.7)	51.0
Unmarried	828 (42.6)	49.1^§^	170 (37.4)	42.1^§^	169 (37.3)	22.3^§^
**Employment status^§§^**						
Working^¶^	1,158 (64.5)	66.9	269 (58.6)	56.9	293 (58.7)	40.2
Not working	682 (35.5)	50.8^§^	194 (41.4)	56.3	193 (41.3)	40.4
**Poverty status** ^¶¶^						
At or above poverty^¶^	1,431 (79.6)	64.8	366 (79.7)	59.4	366 (79.7)	43.1
Below poverty	395 (20.4)	47.8^§^	96 (20.3)	46.3^§^	96 (20.3)	29.2^§^
Area of residence***						
Rural	262 (13.9)	56.8^§^	92 (19.0)	60.9^§^	91 (18.9)	42.9
Nonrural^¶^	1,579 (86.1)	61.9	371 (81.0)	55.6	371 (81.1)	39.7
**Region^†††^**						
Northeast^¶^	379 (18.1)	64.0	75 (13.1)	58.7	75 (13.1)	42.7
Midwest	370 (20.0)	59.5	95 (19.3)	68.8^§^	95 (19.3)	46.8
South	753 (38.0)	59.6	181 (36.9)	50.0^§^	180 (36.8)	34.6^§^
West	339 (23.8)	63.2	112 (30.8)	56.1	112 (30.8)	41.9
**Prenatal insurance status^§§§^**						
Private/Military^¶^	857 (48.7)	67.4	251 (55.2)	64.0	251 (55.3)	46.2
Public	882 (45.8)	56.3^§^	189 (39.9)	49.4*	189 (40.0)	34.7^§^
Uninsured	102 (5.5)	47.9^§^	<30 (—^¶¶¶^)	—^¶¶¶^	<30 (—^¶¶¶^)	—^¶¶¶^
Provider recommendation/offer****						
Offered or referred^¶^	1,294 (71.4)	75.2	346 (74.6)	72.7	286 (62.1)^††††^	57.8
Recommended, no offer or referral	132 (7.3)	50.2^§^	<30 (—^¶¶¶^)	—^¶¶¶^	140 (30.8) ^§§§§^	13.9^§^
No recommendation	388 (21.3)	20.6^§^	95 (20.5)	1.9^§^	34 (7.2) ^¶¶¶¶^	0.0^§^
**No. of provider visits since July 2019**						
None	<30 (—^¶¶¶^)	—^¶¶¶^	N/A	N/A	N/A	N/A
1–5	439 (23.9)	50.6^§^	N/A	N/A	N/A	N/A
6–10	725 (38.7)	63.3	N/A	N/A	N/A	N/A
>10^¶^	652 (36.2)	67.5	N/A	N/A	N/A	N/A
High-risk condition for influenza*****						
Yes^¶^	779 (48.0)	65.9	N/A	N/A	N/A	N/A
No	829 (52.0)	59.1^§^	N/A	N/A	N/A	N/A

**Abbreviations:** N/A = not applicable; Tdap = tetanus toxoid, reduced diphtheria toxoid, and acellular pertussis vaccine.

* Women pregnant any time during October 2019–January 2020 were included in the analyses to assess influenza vaccination coverage for the 2019–20 season. Women who received an influenza vaccination since July 1, 2019, before or during their pregnancy were considered vaccinated.

^†^ Women pregnant any time since August 1, 2019, and who had a live birth were included in the analysis to assess Tdap coverage. Women who received a Tdap vaccination during their recent pregnancy were considered vaccinated.

^§^ ≥5 percentage-point difference compared with referent group.

^¶^ Referent group for comparison within subgroups.

** Race/ethnicity was self-reported. Women identified as Hispanic might be of any race. The “Other” race category included Asians, American Indians/Alaska Natives, Native Hawaiians or other Pacific Islanders, and women who selected “other” or multiple races.

^††^ Excludes one woman who did not report marital status.

^§§^ Women who were employed for wages and self-employed were categorized as working; those who were out of work, homemakers, students, retired, or unable to work were categorized as not working.

^¶¶^ Poverty status was defined based on the reported number of persons living in the household and annual household income, according to U.S. Census poverty thresholds. https://www.census.gov/data/tables/time-series/demo/income-poverty/historical-poverty-thresholds.html.

*** Rurality was defined using ZIP codes where >50% of the population resides in a nonmetropolitan county, a rural U.S. Census tract, or both, according to the Health Resources and Services Administration’s definition of rural population. https://www.hrsa.gov/rural-health/about-us/definition/index.html.

^†††^ *Northeast*: Connecticut, Maine, Massachusetts, New Hampshire, New Jersey, New York, Pennsylvania, Rhode Island, and Vermont. *Midwest*: Illinois, Indiana, Iowa, Kansas, Michigan, Minnesota, Missouri, Nebraska, North Dakota, Ohio, South Dakota, and Wisconsin. *South*: Alabama, Arkansas, Delaware, District of Columbia, Florida, Georgia, Kentucky, Louisiana, Maryland, Mississippi, North Carolina, Oklahoma, South Carolina, Tennessee, Texas, Virginia, and West Virginia. *West*: Alaska, Arizona, California, Colorado, Hawaii, Idaho, Montana, Nevada, New Mexico, Oregon, Utah, Washington, and Wyoming.

^§§§^ Women pregnant on their survey date were asked about current insurance; women who had already delivered were asked about insurance “during your most recent pregnancy.” Women considered to have public insurance selected at least one of the following when asked what kind of medical insurance they had: Medicaid, Medicare, Indian Health Service, state-sponsored medical plan, or other government plan. Women considered to have private/military insurance selected private medical insurance and/or military medical insurance and did not select any type of public insurance.

^¶¶¶^ Estimates with sample size <30 are not reported.

**** Excluded women who did not report having a provider visit since July 2019 (25) for the influenza vaccination coverage analysis; no women were excluded for the Tdap vaccination coverage analysis.

^††††^ Received provider offer/referral for both influenza and Tdap vaccines.

^§§§§^ Received a combination of provider offer/referral, recommendation with no referral, or no recommendation for influenza or Tdap vaccines that does not include receipt of offer/referral for both vaccines or no recommendation received for both vaccines. For example, the respondent might have received an offer/referral for influenza vaccine and a recommendation with no referral for Tdap). If information about provider recommendation for either vaccine was missing, then the respondent was excluded from the analysis (two).

^¶¶¶¶^ Did not receive a provider recommendation for influenza or Tdap vaccine.

***** Conditions other than pregnancy associated with increased risk for serious medical complications of influenza include chronic asthma, a lung condition other than asthma, a heart condition, diabetes, a kidney condition, a liver condition, obesity, or a weakened immune system caused by a chronic illness or by medicines taken for a chronic illness. Women who were missing information (233) were excluded from analysis.

**FIGURE:**
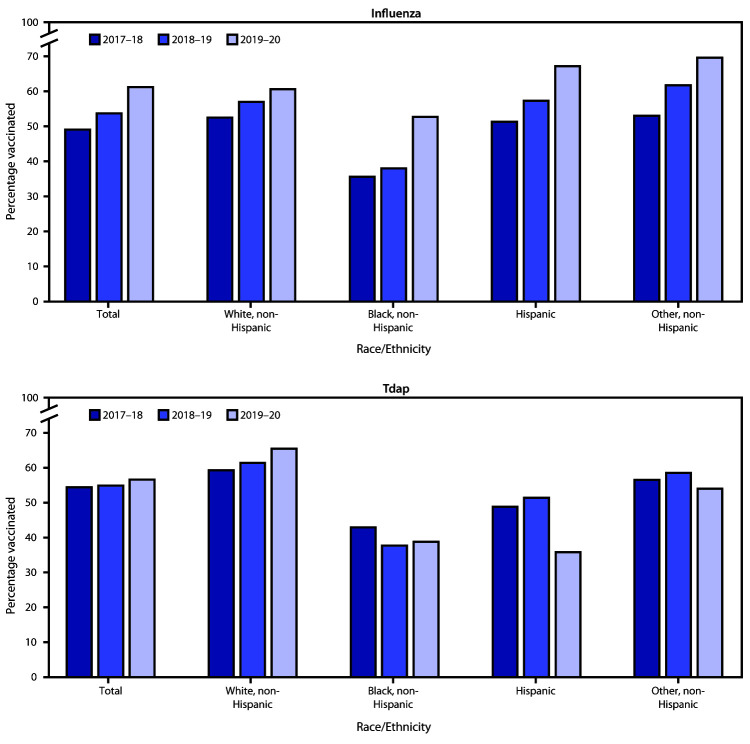
Influenza* and Tdap^†^ vaccination coverage among pregnant women, by race/ethnicity — Internet panel survey, United States, 2017–18^§^ through 2019–20^¶^ influenza seasons **Abbreviation:** Tdap = tetanus toxoid, reduced diphtheria toxoid, and acellular pertussis vaccine. * Women pregnant any time during October 2019–January 2020 were included in the analyses to assess influenza vaccination coverage for the 2019–20 season. Women who received an influenza vaccination since July 1, 2019, before or during their pregnancy, were considered vaccinated. ^†^ Women pregnant any time since August 1, 2019, and had a live birth were included in the analysis to assess Tdap coverage. Women who received a Tdap vaccination during their recent pregnancy were considered vaccinated. ^§^ Kahn KE, Black CL, Ding H, et al. Influenza and Tdap vaccination coverage among pregnant women—United States, April 2018. MMWR Morb Mortal Wkly Rep 2018;67:1055–9. ^¶^ Lindley MC, Kahn KE, Bardenheier BH, et al. Vital signs: burden and prevention of influenza and pertussis among pregnant women and infants—United States. MMWR Morb Mortal Wkly Rep 2019;68:885–92.

Increases in influenza vaccination coverage were observed during 2019–20 for Black women (14.7 percentage points, to 52.7%), Hispanic women (9.9 percentage points, to 67.2%), and women of other races (7.9 percentage points, to 69.6%). Correspondingly, the difference in influenza vaccination coverage between White and Black women decreased from 19 to 8 percentage points from 2018–19 to 2019–20 (Figure). A decrease in Tdap coverage was observed among Hispanic women from 2018–2019 to 2019–2020.

The proportion of women who reported receipt of a provider offer or referral for influenza vaccination was higher among Hispanic women (76.9%) than among White (69.5%) and Black (69.1%) women but was similar to that among women of other races (73.7%). Among women with an offer or referral, influenza vaccination coverage was lower among Black (66.7%) than among White (75.6%) and Hispanic (79.0%) women and women of other races (80.7%) (Table 2). Among women with an offer or referral and 10 or more provider visits, influenza vaccination coverage was 64.3% in Black and 80.5% in White women. Influenza vaccination coverage was similar among White (73.6%) and Black (72.7%) women with an offer or referral and a condition^¶^ (other than pregnancy) that put them at high risk for severe complications from influenza, but among those without high-risk conditions, coverage was lower among Black (62.8%) than among White women (77.4%).

**TABLE 2:** Influenza vaccination coverage among pregnant women* who reported a health care provider offer or referral for vaccination, by selected characteristics, stratified by race/ethnicity^†^ — Internet panel survey, United States, April 2020

Characteristic	All women		White, non-Hispanic		Black, non-Hispanic		Hispanic		Other, non-Hispanic	
	No. (weighted %)	% (weighted) vaccinated	No. (weighted %)	% (weighted) vaccinated	No. (weighted %)	% (weighted) vaccinated	No. (weighted %)	% (weighted) vaccinated	No. (weighted %)	% (weighted) vaccinated
**Total**	1,294 (100)	75.2	613 (100)	75.6	216 (100)	66.7	329 (100)	79.0	136 (100)	80.7
**Age group (yrs)**										
18–24	438 (24.1)	67.1^§^	132 (21.4)	64.2^§^	108 (29.9)	65.6	151 (29.0)	71.5^§^	47 (11.2)	76.6
25–34	611 (55.8)	77.7	333 (57.9)	79.5	81 (52.4)	64.0	137 (52.4)	83.8^§^	60 (61.6)	79.5
35–49	245 (20.1)	77.8	148 (20.7)	76.8	<30 (—**)	—**	41 (18.7)	77.5	<30 (—**)	—**
**Education**										
High school diploma or less	273 (20.0)	64.2^§^	130 (21.0)	57.1^§^	52 (20.2)	55.4^§^	75 (21.7)	81.3	<30 (—**)	—**
Some college, no degree	194 (15.2)	65.3^§^	78 (13.5)	62.1^§^	39 (19.1)	69.4	53 (15.2)	69.8^§^	<30 (—**)	—**
College degree (2- or 4-year)	521 (41.4)	81.0	251 (41.6)	84.6	86 (41.6)	69.9	123 (38.5)	80.9	61 (48.1)	85.4
More than college degree^¶^	306 (23.4)	80.6	154 (23.9)	84.0	39 (19.1)	69.0	78 (24.5)	79.8	35 (27.8)	84.5
**Marital status** ^††^										
Married^¶^	757 (61.2)	81.0	418 (68.0)	80.6	84 (43.9)	79.5	169 (57.4)	81.1	86 (73.4)	85.2
Unmarried	537 (38.8)	66.0^§^	195 (32.0)	65.2^§^	132 (56.1)	56.6^§^	160 (42.6)	76.2	50 (26.6)	68.6^§^
**Employment status** ^§§^										
Working^¶^	847 (67.3)	79.2	410 (66.1)	79.7	147 (72.8)	70.6	206 (65.5)	82.1	84 (66.8)	89.5
Not working	446 (32.7)	66.9^§^	203 (33.9)	67.8^§^	68 (27.2)	55.2^§^	123 (34.5)	73.3^§^	52 (33.2)	63.0^§^
**Poverty status** ^¶¶^										
At or above poverty^¶^	1032 (81.3)	78.3	511 (83.4)	80.0	150 (72.9)	69.5	258 (81.7)	79.7	113 (88.0)	82.3
Below poverty	253 (18.7)	62.1^§^	100 (16.6)	53.5^§^	63 (27.1)	59.4^§^	68 (18.3)	77.2	<30 (—**)	—**
Area of residence***										
Rural	174 (13.1)	72.2	105 (17.5)	70.3^§^	<30 (—**)	—**	<30 (—**)	—**	<30 (—**)	—**
Nonrural^¶^	1,120 (86.9)	75.6	508 (82.5)	76.8	189 (88.5)	65.8	301 (93.0)	79.2	122 (91.6)	80.9
**Region** ^†††^										
Northeast^¶^	276 (18.8)	78.2	154 (22.1)	77.3	37 (16.0)	71.4	71 (17.2)	84.6	<30 (—**)	—**
Midwest	255 (19.8)	72.3^§^	142 (23.4)	71.6^§^	39 (18.7)	62.0^§^	50 (15.2)	79.7	<30 (—**)	—**
South	520 (37.3)	74.2	217 (33.2)	74.5	125 (53.6)	70.6	122 (34.0)	77.4^§^	56 (34.0)	76.3
West	243 (24.1)	76.7	100 (21.3)	80.0	<30 (—**)	—**	86 (33.5)	77.5^§^	42 (41.8)	82.1
**Prenatal insurance status^§§§^**										
Private/Military^¶^	631(50.9)	79.8	359 (58.1)	82.1	80 (41.2)	68.6	119 (39.8)	80.1	73 (65.6)	83.0
Public	608 (44.9)	70.5^§^	229 (37.8)	65.6^§^	125 (54.3)	67.3	196 (56.1)	77.9	58 (31.4)	77.7^§^
Uninsured	55 (4.2)	70.1^§^	<30 (—**)	—**	<30 (—**)	—**	<30 (—**)	—**	<30 (—**)	—**
**No. of provider visits since July 2019**										
1–5	257 (19.7)	70.2^§^	111 (18.0)	65.5^§^	36 (17.2)	61.8	77 (23.5)	80.5	33 (23.9)	74.1^§^
6–10	522 (39.8)	76.2	248 (40.5)	75.2^§^	89 (39.5)	71.4^§^	133 (39.7)	80.8	52 (36.8)	79.2^§^
>10^¶^	515 (40.5)	76.6	254 (41.4)	80.5	91 (43.4)	64.3	119 (36.8)	76.1	51 (39.4)	86.3
**High-risk condition for influenza** ^¶¶¶^										
Yes^¶^	606 (51.7)	76.8	254 (44.3)	73.6	112 (59.2)	72.7	183 (65.5)	82.5	57 (40.0)	86.2
No	546 (48.3)	75.8	314 (55.7)	77.4	74 (40.8)	62.8^§^	92 (34.5)	81.2	66 (60.0)	78.9^§^

* Women pregnant any time during October 2019–January 2020 were included in the analyses to assess influenza vaccination coverage for the 2019–20 season. Women who received an influenza vaccination since July 1, 2019, before or during their pregnancy were considered vaccinated.

^†^ Race/ethnicity was self-reported. Women identified as Hispanic might be of any race. The “Other” race category included Asians, American Indians/Alaska Natives, Native Hawaiians or Other Pacific Islanders, and women who selected “other” or multiple races.

^§^ ≥5 percentage-point difference compared with referent group.

^¶^ Referent group for comparison within subgroups.

** Estimates with sample size <30 are not reported.

^††^ Excludes one woman who did not report marital status.

^§§^ Women who were employed for wages and self-employed were categorized as working; those who were out of work, homemakers, students, retired, or unable to work were categorized as not working.

^¶¶^ Poverty status was defined based on the reported number of persons living in the household and annual household income, according to U.S. Census poverty thresholds. https://www.census.gov/data/tables/time-series/demo/income-poverty/historical-poverty-thresholds.html.

*** Rurality was defined using ZIP codes where >50% of the population resides in a nonmetropolitan county, a rural U.S. Census tract, or both, according to the Health Resources and Services Administration’s definition of rural population. https://www.hrsa.gov/rural-health/about-us/definition/index.html.

^†††^ *Northeast*: Connecticut, Maine, Massachusetts, New Hampshire, New Jersey, New York, Pennsylvania, Rhode Island, and Vermont. *Midwest*: Illinois, Indiana, Iowa, Kansas, Michigan, Minnesota, Missouri, Nebraska, North Dakota, Ohio, South Dakota, and Wisconsin. *South*: Alabama, Arkansas, Delaware, District of Columbia, Florida, Georgia, Kentucky, Louisiana, Maryland, Mississippi, North Carolina, Oklahoma, South Carolina, Tennessee, Texas, Virginia, and West Virginia. *West*: Alaska, Arizona, California, Colorado, Hawaii, Idaho, Montana, Nevada, New Mexico, Oregon, Utah, Washington, and Wyoming. ^§§§^ Women pregnant on their survey date were asked about current insurance; women who had already delivered were asked about insurance “during your most recent pregnancy.” Women considered to have public insurance selected at least one of the following when asked what kind of medical insurance they had: Medicaid, Medicare, Indian Health Service, state-sponsored medical plan, or other government plan. Women considered to have private/military insurance selected private medical insurance and/or military medical insurance and did not select any type of public insurance.

^¶¶¶^ Conditions other than pregnancy associated with increased risk for serious medical complications of influenza include chronic asthma, a lung condition other than asthma, a heart condition, diabetes, a kidney condition, a liver condition, obesity, or a weakened immune system caused by a chronic illness or by medicines taken for a chronic illness. Women who were missing information (142) were excluded from analysis.

Receipt of a provider offer or referral for Tdap was lower among Black (55.7%) than among Hispanic women (66.6%), women of other races (71.3%), and White women (81.0%). Among those with a provider offer or referral for Tdap vaccination, Tdap coverage was lowest for Hispanic women (52.5%), followed by Black women (64.7%), women of other races (73.1%), and White women (77.5%).

## Discussion

Findings from this survey indicate that approximately 40% of pregnant women do not receive influenza and Tdap vaccines, leaving themselves and their infants more vulnerable to influenza and pertussis infection, with potential serious complications including hospitalization and death (*4*). Although influenza vaccination coverage remains suboptimal, an increase in coverage was observed during 2019–20. The overall increase was driven by increased vaccination coverage among Black and Hispanic women and those of other races. Higher vaccination coverage was observed among women who received a provider offer or referral or a recommendation alone (*4*), indicating increased acceptance of vaccination overall. However, despite approximately 70% of Black and White women receiving a provider offer or referral for influenza vaccination, Black women were still less likely to be vaccinated than White women. Factors including negative attitudes and beliefs about vaccines, less knowledge about and access to vaccines, and a lack of trust in health care providers and vaccines has been shown to contribute to lower vaccination rates in Black adults (*6*,*7*). Provider offers or referrals for vaccination, in combination with culturally competent conversations with patients, could increase vaccination coverage among pregnant women in all racial/ethnic groups and reduce disparities (*8*).

Approximately 20% of pregnant women reported not receiving a provider recommendation for vaccination. This circumstance might be partly attributable to differences in perception of a provider recommendation between patients and providers. One study indicated that providers might believe they are giving a recommendation for vaccination, but it might not be remembered by patients (*9*). Differences by patient race/ethnicity in reported vaccination offers might result from provider-patient communication problems or reflect deficits in quality of care provided to some minority patients (*10*). CDC has resources to assist providers in effectively communicating the importance of vaccination, such as sharing specific reasons that recommended vaccines are right for the patient and highlighting positive experiences with vaccines (personal or clinical).** In addition, the American College of Obstetricians and Gynecologists has an immunization toolkit^††^ that includes communication strategies for providers.

The findings in this report are subject to at least three limitations (*5*). First, this was a nonprobability sample, and results might not be generalizable to all pregnant women in the United States. Second, vaccination status was self-reported and might be subject to recall or social desirability bias. Finally, Tdap coverage estimates are subject to uncertainty, given the small sample size and exclusion of 12.9% of women with unknown Tdap vaccination status. Despite these limitations, Internet panel surveys are a useful assessment tool for timely evaluation of routine maternal vaccination coverage.

Despite ACIP recommendations and an increase of approximately 12 percentage points in influenza vaccination since the 2017–18 season, maternal vaccination with influenza and Tdap vaccines is suboptimal, and missed opportunities to vaccinate are common. Although racial/ethnic disparities in vaccination persist, the magnitude in coverage differences were reduced in the 2019–20 influenza season as a result of increased vaccination coverage in Black, Hispanic, and other race women. Increases or decreases in vaccination coverage observed in this survey should be compared with information from other data sources and additional survey years. Racial disparities in vaccination coverage could decrease further with consistent provider offers or referrals for vaccination, in combination with culturally competent conversations with patients^§§^ (*8*,*9*).

**Summary**

**What is already known about this topic?**

Maternal vaccination with influenza and tetanus toxoid, reduced diphtheria toxoid, and acellular pertussis (Tdap) vaccines can decrease the risk for severe influenza and pertussis among pregnant women and their infants; racial/ethnic coverage disparities exist.

**What is added by this report?**

During 2019–20, 61.2% of pregnant women received influenza vaccination, 56.6% received Tdap during pregnancy, and 40.3% received both vaccines. Influenza vaccination coverage among Black and Hispanic women increased, yet disparities persisted; Tdap vaccination increased among Black women but decreased in Hispanic women compared with 2018–19.

**What are the implications for public health practice?**

Additional interventions to encourage consistent provider offers or referrals for influenza and Tdap vaccination and culturally competent conversations with patients are needed to address racial disparities in maternal vaccination.

## Footnotes

* Pregnant women were recruited from a large, pre-existing, opt-in Internet panel of the general population, a panel operated by Dynata (https://www.dynata.com).

† An opt-in Internet panel survey is a nonprobability sampling survey. The denominator for a response rate calculation cannot be determined because no sampling frame with a selection probability is involved at the recruitment stage. Instead, the survey cooperation rate (the percentage interviewed among all eligible persons contacted) is provided.

§ Because the opt-in Internet panel sample is not probability-based, no statistical tests were performed. Additional information on obstacles to inference in nonprobability samples is available at https://www.aapor.org/AAPOR_Main/media/MainSiteFiles/NPS_TF_Report_Final_7_revised_FNL_6_22_13.pdf and https://www.aapor.org/getattachment/Education-Resources/For-Researchers/AAPOR_Guidance_Nonprob_Precision_042216.pdf.aspx. Although the estimates reported here have variance, there has been no attempt to quantify the size of the variance.

¶ Conditions other than pregnancy associated with increased risk for serious medical complications of influenza include chronic asthma, a lung condition other than asthma, a heart condition, diabetes, a kidney condition, a liver condition, obesity, or a weakened immune system caused by a chronic illness or by medicines taken for a chronic illness.

** https://www.cdc.gov/vaccines/hcp/adults/for-practice/standards/recommend.html.

†† https://www.acog.org/programs/immunization-for-women/provider-tools.

§§ https://www.thecommunityguide.org/topic/vaccination.
